# Mobile Phone Base Station Exposure and Symptoms

**DOI:** 10.1289/ehp.10771

**Published:** 2008-02

**Authors:** Martin Röösli, Anke Huss

**Affiliations:** Institute of Social and Preventive Medicine, University of Bern, Bern, Switzerland, E-mail: Roeoesli@ispm.unibe.ch

Eltiti et al. (2007) reported elevated levels of arousal when electromagnetic-hypersensitive subjects were exposed to a UMTS (universal mobile telecommunications system) mobile phone base station signal of 10 mW/m^2^. Based on their statistical analysis, they concluded that this observation was likely to be due to the effect of order of exposure rather than the exposure itself. In our view, however, a critical review of their data suggests a different conclusion.

First of all, Eltiti et al. (2007) hypothesized that

Sensitive participants would report more symptoms and lower levels of well-being during GSM [global system for mobile communication] and UMTS exposure compared to sham.

When dealing with a directional hypothesis, a one-sided statistical test is indicated. According to a one-sided statistical test, differences between sham and UMTS exposure for sensitive subjects regarding anxiety (*t*-value = 2.89) and tension (*t*-value = 2.94) are significant, even after applying a Bonferroni correction.

An arguable issue is whether Bonferroni correction should be applied in the first place. The trial was designed to replicate previous findings from a Dutch study (Zwamborn et al. 2003).

Many statisticians may point out that multiple end point correction is not needed under these circumstances. Definitely, a Bonferroni correction, as used in the context of the trial by Eltiti et al. (2007), is too conservative when measuring several symptoms that are very likely to be correlated. The correlation between the outcomes should be taken into account in the multiple end point correction. As a consequence, the reference *t*-values would be lower, again yielding the conclusion that anxiety and tension are correlated with UMTS exposure.

It is unfortunate that the exposure order among the three conditions was not counterbalanced. As Eltiti et al. (2007) reported, this unbalanced design led to additional variation in the data. We therefore cannot understand why the authors did not include the order of exposure conditions as a factor in their statistical model. Instead, they presented a between-subjects comparison stratified by order [see Table 3 in Eltiti et al. (2007)]. It is true that the differences between sham and UMTS did not reach statistical significance in any of the three sessions. However, it is striking that in each of the three sessions, the arousal score of sensitive individuals was higher for the UMTS condition compared to sham. Pooling the three sessions together would yield a significant difference between sham and UMTS (*t*-test; *p* = 0.02). Likewise, a meta-regression of the data from their Table 3 confirms that order (*p* = 0.043) and exposure condition (*p* = 0.076) are important factors and should have been considered in the original model.

Finally, given the fact that Eltiti et al. (2007) observed a few more borderline significant effects and that the targeted sample size was not achieved, one would expect a critical discussion about the power of the study, which the authors did not provide.

In summary, a more careful data analysis yields significantly different tension, arousal, and anxiety scores between sham and UMTS exposure status for sensitive subjects. It seems unlikely that these differences are solely due to order of exposure, as argued by Eltiti et al. (2007) .

We think that results from this study should be interpreted with more caution. Certainly, an association between low-level short-term UMTS mobile phone base station exposure and symptoms is unexpected and contradicts a previous study (Regel et al. 2006). This issue merits further clarification.
